# Source Field Plate Incorporated Monolithic Inverters Composed of GaN-Based CMOS-HEMTs with Double-2DEG Channels and Fin-Gated Multiple Nanochannels

**DOI:** 10.3390/ma19061209

**Published:** 2026-03-19

**Authors:** Hong-You Chen, Hsin-Ying Lee, Hao Lee, Yuh-Renn Wu, Ching-Ting Lee

**Affiliations:** 1 Institute of Microelectronics, Department of Electrical Engineering, National Cheng Kung University, Tainan 701, Taiwan; xxxxx6712@gmail.com; 2 Department of Photonics, National Cheng Kung University, Tainan 701, Taiwan; 3 Graduate Institute of Photonics and Optoelectronics, National Taiwan University, Taipei 10617, Taiwan; imhoward26@gmail.com (H.L.); yrwu@ntu.edu.tw (Y.-R.W.); 4 Department of Electrical Engineering, Yuan Ze University, Taoyuan 320, Taiwan

**Keywords:** GaN-based complementary metal–oxide–semiconductor high-electron-mobility transistors, double-2DEG channels, fin-gated multiple nanochannels, monolithic inverter, source field plate

## Abstract

**Highlights:**

- Monolithic inverters of CMOS-HEMTs consisting of D- and E-mode GaN-based devices were fabricated.
- Characteristics of GaN-based MOS-HEMTs were influenced by the source field plate length.
- Double-2DEG channels and fin-gated multiple nanochannels enhanced the device’s characteristics.

**Abstract:**

In this study, enhancement- and depletion-mode (E- and D-mode) GaN-based 120 nm-wide fin-gated multiple nanochannel metal–oxide–semiconductor high-electron-mobility transistors (MOS-HEMTs) were manufactured on the epitaxial Al_0.83_In_0.17_N/GaN/Al_0.18_Ga_0.82_N/GaN two-dimensional electron gas (2DEG) channel layers grown on Si substrates using a metal-organic chemical vapor deposition system. The oxide layer grown directly by the photoelectrochemical oxidation method was used as the gate oxide layer in D-mode MOS-HEMTs. Furthermore, E-mode MOS-HEMTs used ferroelectric stacked LiNbO_3_/HfO_2_/Al_2_O_3_ layers as the gate oxide layers. The 120 nm-wide multiple nanochannels and various-length source field plates (SFPs) were fabricated and incorporated into monolithic complementary MOS-HEMTs (CMOS-HEMTs) consisting of D- and E-mode MOS-HEMTs. The resulting monolithic unskewed inverter was achieved by modulating the drain-source current of the D-mode MOS-HEMTs. The noise low margin of 2.03 V and noise high margin of 2.10 V of the unskewed monolithic inverter were obtained. From the dynamic experimental results, the rising time and falling time of the unskewed monolithic inverter were 4.9 μs and 3.2 μs, respectively. The breakdown voltage could be improved by incorporating an SFP. When the SFP edge was located at the center between the gate electrode and the drain electrode, the maximum breakdown voltage of 855 V was obtained.

## 1. Introduction

Recently, owing to significant advances in epitaxial growth, manufacturing, and design techniques, gallium nitride (GaN)-based semiconductors have been successfully employed to fabricate various GaN-based high-electron-mobility transistors (HEMTs) and to apply them in practical systems [1,2,3,4]. To increase the drain-source current (I_DS_) and thus boost device power, one effective method is to simultaneously improve both the sheet electron density and the electron mobility in the two-dimensional electron gas (2DEG) channel induced at the interface between the hetero-structured AlGaN and GaN layers. Among them, one method is to use complex epitaxial growth techniques to grow an AlGaN barrier layer with a higher Al content on the GaN channel layer. While this method can increase the sheet electron density in the 2DEG channel, it also reduces its electron mobility [5]. In recent years, multiple 2DEG channels have been epitaxially grown and used to fabricate depletion-mode (D-mode) and enhancement-mode (E-mode) GaN-based planar HEMTs, thereby overcoming the challenges [6,7,8]. Because the lattice-matched hetero-structured 2DEG channel structures can mitigate the failure induced by the inverse piezoelectric effect of the strain, the lattice-matched AlInGaN/GaN hetero-structured layers were grown and utilized for fabricating GaN-based HEMTs [9,10]. The epitaxial layers of the double-2DEG channels of the Al_0.83_In_0.17_N/GaN/Al_0.18_Ga_0.82_N/GaN were grown on silicon (Si) substrates using a metal-organic chemical vapor deposition system (AIXTRON Group, Herzogenrath, Germany) and were used to fabricate D-mode GaN-based metal-oxide-semiconductor HEMTs (MOS-HEMTs) previously [11,12]. Due to the superior gate control capability of the fin-gate structure, GaN-based MOS-HEMTs with fin-gated multiple nanochannels exhibited higher performance than that of the planar devices [12,13,14]. Moreover, field plate engineering has been employed to improve breakdown characteristics, alleviate current collapse, and mitigate dynamic degradation by tailoring the electric field profiles and potential lines [15,16,17]. The literature already contained a considerable number of planar GaN-based D- and E-mode HEMTs that utilized various structures and manufacturing processes. Even fin-gated multiple nanochannel ones were recently developed and investigated. However, those devices were almost manufactured using a single-2DEG channel epitaxial structures. In this study, double-2DEG channels of the epitaxial Al_0.83_In_0.17_N/GaN/Al_0.18_Ga_0.82_N/GaN layers were grown. The D- and E-mode GaN-based fin-gated multiple nano MOS-HEMTs were fabricated using the epitaxial layers. Furthermore, various source field plates were introduced in the D- and E-mode devices. In this study, by integrating SFP-incorporated D- and E-mode MOS-HEMTs with double-2DEG channels and fin-gated multiple nanochannels to form complementary MOS-HEMTs (CMOS-HEMTs), the resulting monolithic inverters were also fabricated, measured, and analyzed. Furthermore, to explore the affected functions and characteristics, various-length SFPs were designed and manufactured.

## 2. Monolithic Inverters Fabrication Process

**Figure 1:**
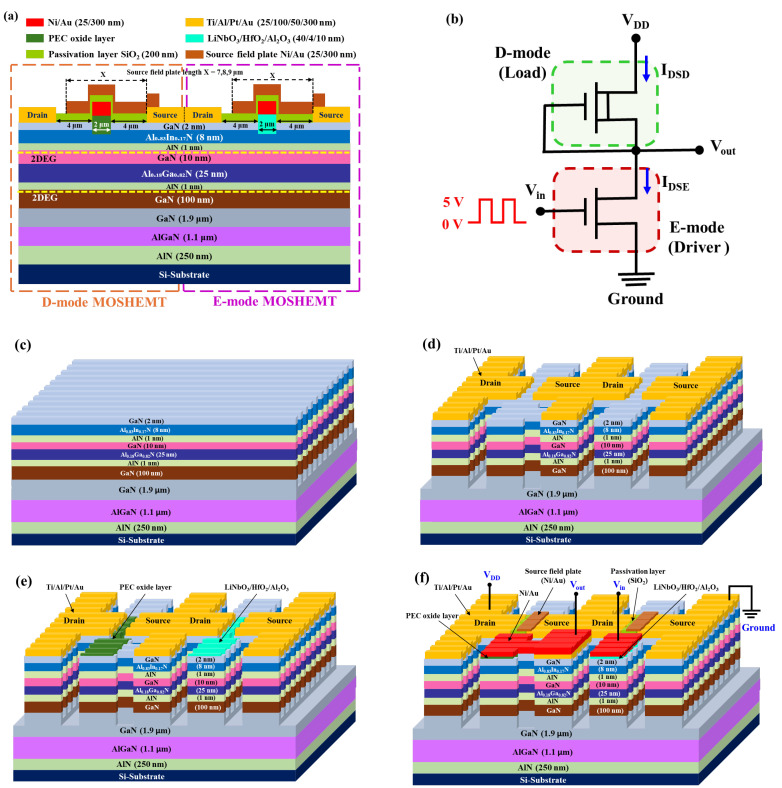
(**a**) Cross-sectional configuration and (**b**) equivalent circuit of source field plate incorporated monolithic inverters, and three-dimensional configuration of (**c**) multiple nanochannel structure, (**d**) source and drain electrode’s structure, (**e**) gate oxide layers, and (**f**) source field plate incorporated monolithic inverters.

The schematic configuration of the cross-sectional and the associated equivalent circuit of the monolithic inverters were respectively illustrated in Figure 1a,b. The E-mode device and the D-mode device worked in driver and load roles, respectively, for the monolithic inverter. The epitaxial layers were grown on a Si substrate using a metal-organic chemical deposition system. The used Si substrates were 6-inch and 1000 μm-thick Boron-doped p-type Si wafers with [111]-orientation and resistivity of 3 Ω-cm. The wafers were manufactured by Wafer Works Company. The epitaxial growth processes, high-resolution transmission electron microscopy, and simulated band diagram and electron concentration distribution of the epitaxial layers were reported previously [11]. The double-2DEG channels were formed by the band-discontinued lattice-matched Al_0.83_In_0.17_N (8 nm)/GaN (10 nm) layers and the polarized Al_0.18_Ga_0.82_N (25 nm)/GaN (100 nm) layers, respectively. The double-2DEG channels are marked in Figure 1a. Using a Hall measurement at room temperature, the total sheet electron density and the equivalent electron mobility were measured to be 1.1 × 10^13^ cm^−2^ and 1770 cm^2^/Vs, respectively.

After spring positive photoresist CSAR62 onto the epitaxial wafer, an electron-beam lithography system (ELS-BODEN 50, ELIONIX Inc., Tokyo, Japan) was used to pattern parallel straight multiple nanochannels. The channel width and channel spacing were 120 nm and 880 nm, respectively. When a 50 nm-thick Ni mask was deposited using an electron beam evaporator, the multiple nanochannels were then fabricated using an inductively coupled plasma system with a mixed boron trichloride and chlorine etchant gas. By removing the Ni metal mask using a hydrochloric acid (HCl) chemical solution, the resulting multiple nanochannels were illustrated in Figure 1c. An electron beam evaporator was used to deposit a 50 nm-thick Ni metal on the window of mesa isolation regions patterned by a standard photolithography method. After a lift-off process of the undesired metal, the mesa isolation regions were formed by using the mixed etchant gases under the protection of the patterned Ni metal mask. After the patterned Ni metal mask was removed using a HCl chemical solution, the windows of the source region and drain region were opened using a standard photolithography method. Ti/Al/Pt/Au (25/100/50/300 nm) metals for the source and drain electrodes of D- and E-mode MOS-HEMTs were deposited using an electron-beam evaporator and then annealed in a nitrogen ambient at 900 °C for 30 s. The source and drain electrodes were separated by 10 μm. Figure 1d shows the resulting figuration. Using a standard photolithography system to open 2 μm-wide straight gate windows of the D-mode device. The gate oxide layer and the gate-recessed structure were simultaneously obtained using the previously reported photoelectrochemical (PEC) oxidation method [18,19]. This directly grown gate oxide layer surrounded the top and two sidewalls of the nanochannels, forming fin-gated channels for the D-mode MOS-HEMTs. By controlling the PEC process time, the recessed gate depth of the D-mode MOS-HEMT could be adjusted, thereby adjusting the drain-source current to the required value. In this study, the depths were 6 nm, 7 nm, and 8 nm, respectively. To stabilize the gate oxide layers, they were converted into mixed crystalline α-Al_2_O_3_ and β-Ga_2_O_3_ materials by annealing them in an oxygen ambient at 700 °C for 2 h [20]. The distance between the gate electrode and the source electrode, and the distance between the gate electrode and the drain electrode were 4 μm, respectively. To fabricate the gate of E-mode MOS-HEMTs, the 2 μm-wide straight windows were opened using a standard photolithography system. After the gate-recessed regions with a depth of 6 nm were created using the previously reported PEC etching method [21], an atomic layer deposition system was utilized to first deposit Al_2_O_3_ tunneling layer (10 nm) and HfO_2_ charge storage layer (4 nm), and then Krypton fluoride pulsed laser system was used to deposit LiNbO_3_ ferroelectric blocking layer (40 nm) sequentially. The ferroelectric stacked LiNbO_3_/HfO_2_/Al_2_O_3_ gate oxide layers surrounded the top and two sidewalls of the nanochannels, forming fin-gated channels for the E-mode MOS-HEMTs. To partially compensate for the electrons in the 2DEG channels, the strong (006) crystalline ferroelectric phase of the LiNbO_3_ film, which has the upward C^+^ domain polarization and opposite to the polarization direction of the 2DEG channels, was obtained by annealing in an oxygen ambient at 600 °C for 30 min. The X-ray diffraction pattern and the vertical piezoelectric force microscopy images of the LiNbO_3_ films were reported previously [22]. The resulting configuration was illustrated in Figure 1e. Using a standard photolithography method to open the windows for the gate regions of D- and E-mode MOS-HEMTs and the connection electrodes among the gate and source of the D-mode MOS-HEMTs and the drain of the E-mode MOS-HEMT, Ni/Au (25/200 nm) metals were deposited using an electron-beam evaporator. To incorporate various SFPs onto both the D-mode device and E-mode device, a SiO_2_ passivation layer (200 nm) was deposited on the photolithographic pattern windows between the source and drain electrodes using a plasma-enhanced chemical deposition system. After patterning the SFP region, Ni/Au (25/300) metals were deposited, and the unnecessary Ni/Au metals were lifted off. The SFPs were incorporated into the D-mode device and E-mode device of the monolithic inverters. The resulting configuration was shown in Figure 1f. To explore the dependence of the drain-source breakdown voltage on the edge location of SFP, 7, 8, and 9 μm-length SFPs were designed and fabricated. In this study, the distance between the source and drain electrodes was 10 μm, and the 2 μm-wide gate electrode was located in the center between them. Therefore, the edges of 7, 8, and 9 μm-length SFPs were located near the gate electrode, in the center between the gate electrode and drain electrode, and near the drain electrode. In Figure 1f, in addition to showing that the Ni/Au electrode connected the gate and source of the D-mode device to the drain of the E-mode device, the input voltage (V_in_), output voltage (V_out_), and ground of the monolithic inverter were also indicated.

## 3. Experimental Results and Discussion

**Figure 2:**
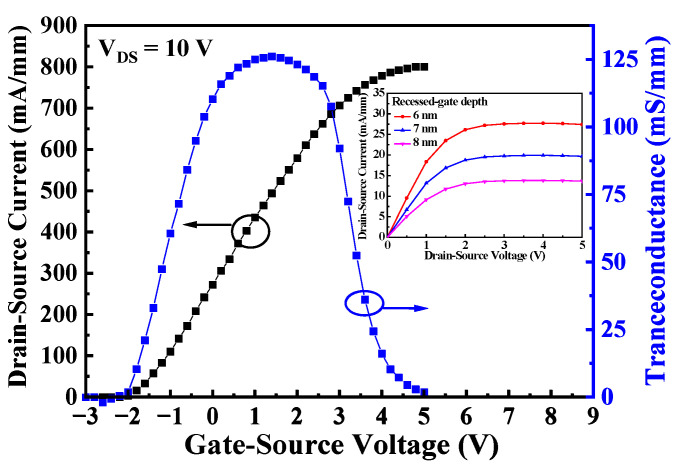
Drain-source current and transconductance as a function of gate-source voltage of the D-mode MOS-HEMTs. Under V_GS_ of 0 V, the inset figure was associated with I_DSD_—V_DSD_ characteristics with recessed-gate depths of 6, 7, and 8 nm.

**Figure 3:**
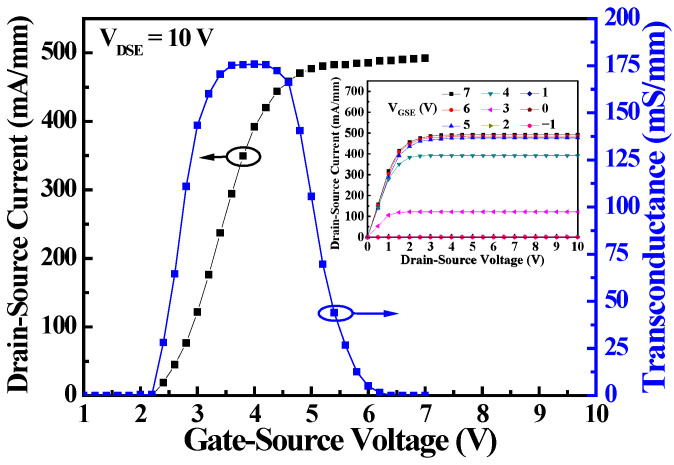
Drain-source current and transconductance as a function of gate-source voltage of E-mode MOS-HEMTs. The inset figure was associated with I_DSE_—V_DSE_ characteristics.

The direct-current (DC) characteristics of the D- and E-mode MOS-HEMTs in the monolithic inverter were respectively measured using an Agilent 4156C semiconductor parameter analyzer. Figure 2 shows the drain-source current (I_DSD_)—gate-source voltage (V_GSD_) characteristics and the transconductance (g_mD_)—V_GSD_ characteristics of the D-mode MOS-HEMT with a recessed-gate depth of 6 nm. The drain-source current was the measured drain-source current normalized with the effective channel width. When the threshold voltage was defined as the V_GSD_ corresponding to the I_DSD_ of 0.1 mA/mm, the threshold voltage (V_thD_) of the D-mode MOS-HEMTs was approximately −1.9 V. The maximum transconductance (g_mD,max_) was approximately 125 mS/mm. When the V_GSD_ was 5 V, the I_DSD_ of the D-mode MOS-HEMTs operating at V_DSD_ of 5 V was 801.3 mA/mm. The inset of Figure 2 shows the associated I_DSD_—drain-source voltage (V_DSD_) characteristics of the D-mode MOS-HEMTs with recessed-gate depths of 6, 7, and 8 nm. Under the V_DSD_ = 5 V and V_GSD_ = 0 V, the D-mode MOS-HEMTs with recessed-gate depths of 6 nm, 7 nm, and 8 nm exhibited I_DSD_ of 27.3 mA/mm, 19.6 mA/mm, and 13.6 mA/mm, respectively. After the E-mode MOS-HEMT was initialized at a gate-source voltage (V_GSE_) of 12 V for 10 ms, Figure 3 shows the drain-source current (I_DSE_)—V_GSE_ characteristics and the transconductance (g_mE_)—V_GSE_ characteristics, and the inset shows the I_DSE_—V_DSE_ characteristics. The threshold voltage (V_thE_) and maximum transconductance (g_mE,max_) of the E-mode MOS-HEMTs were approximately 2.3 V and 125.9 mS/mm, respectively. Under the operation of V_DSE_ = 5 V and V_GSE_ = 5 V, the I_DSE_ was approximately 492.1 mA/mm.

**Figure 4:**
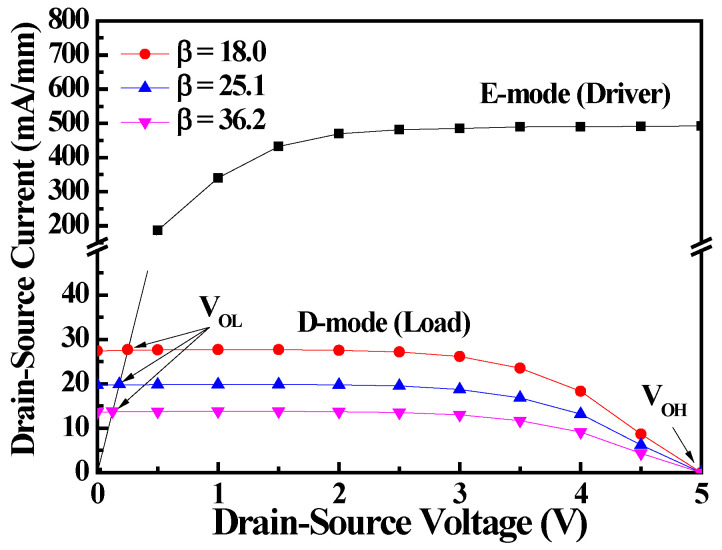
Load lines of monolithic inverters with various β values.

As the equivalent circuit of the monolithic inverter shown in Figure 1b, when the input voltage (V_in_) with a 5-V-amplitude pulsed-rectangular waveform is biased to the gate of the E-mode MOS-HEMT, the output voltage (V_out_) is obtained from the drain of the E-mode MOS-HEMT. To investigate the performance of the monolithic inverters, D-mode MOS-HEMTs with gate-recessed depths of 6 nm, 7 nm, and 8 nm were fabricated. When the monolithic inverter operated at V_DD_ of 5 V and V_in_ of 5 V, because the gate and source of the D-mode MOS-HEMT were shorted (i.e, V_GSD_ = 0 V), Figure 4 shows the load line curves of the monolithic inverters with the above-mentioned three D-mode MOS-HEMTs. The I_DSE_ curve of the E-mode MOS-HEMT operated at V_GSE_ = 5 V was also shown in Figure 4. To distinguish the D-mode MOS-HEMTs from the monolithic inverters, the drain-source current ratio (I_DSE_/I_DSD_) of the E- and D-mode MOS-HEMTs was defined as β. For the D-mode MOS-HEMTs fabricated with recessed-gate depths of 6 nm, 7 nm, and 8 nm, the associated β value was approximately 18.0, 25.1, and 36.2, respectively. As shown in Figure 4, the I_DSE_ curve intersected the load line curves and created the intersection points that are the low output voltages (V_OL_) of the monolithic inverters. The V_OL_ was 0.13 V, 0.08 V, and 0.07 V as β = 18.0, 25.1, and 36.2, respectively. The high output voltage (V_OH_) of the monolithic inverters was defined as V_out_ = V_DD_ = 5 V. It was observed that the V_OL_ decreased with increasing β value. By changing the amplitude of V_in_ from 0 to 5 V, the associated output voltage swing (V_OS_ = V_DD_ − V_OL_) was swept to 4.87 V, 4.91 V, and 4.93 V, respectively. The V_OS_ increased with increasing the β value.

**Figure 5:**
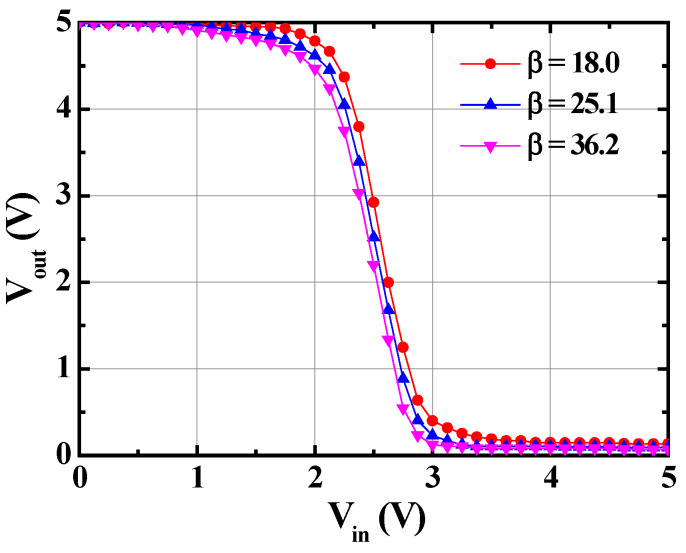
Static voltage transfer characteristics of monolithic inverters with various β values.

**Figure 6:**
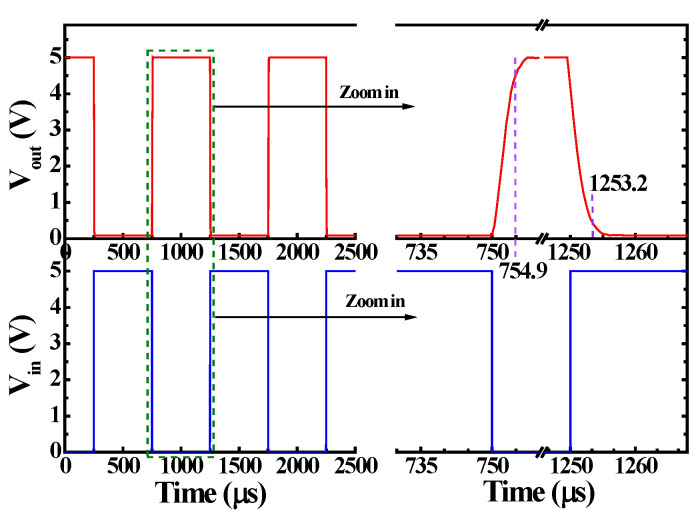
V_out_—V_in_ dynamic characteristics of unskewed monolithic inverter.

The static voltage transfer characteristics (SVTC) of the monolithic inverters with different β values are shown in Figure 5. The low input voltage (V_IL_) and high input voltage (V_IH_) were defined as the V_in_ and V_out_, corresponding to a tangent slope of −1 of the SVTC. Table 1 lists the calculated data. Except for the V_OH_ that remained at 5.0 V, the values of the other V_IL_, V_IH_, and V_OL_ decreased as the β value increased. In addition, to investigate the tolerable noise variation range within which the monolithic inverters with different values would not produce logic errors, the noise low margin (NM_L_ = V_IL_ − V_OL_) and noise high margin (NM_H_ = V_OH_ − V_IH_) were evaluated and are listed in Table 1. The NM_L_ and NM_H_ values were increased with an increase in β. When V_out_ = V_DD_/2 = 2.5 V, the V_in_ was 2.61 V, 2.51 V, and 2.45 V as β = 18.0, 25.1, and 36.2, respectively. In general, when V_out_ was V_DD_/2, the monolithic inverter exhibited optimal unskewed behaviors when V_in_ was also V_DD_/2. It could be deduced that the optimal unskewed inverter could be achieved as β of 25.1. Comparing the NM_H_ and NM_L_ of the monolithic inverters with β of 18.0, 25.1, and 36.2, the monolithic inverter with β of 18.0 has greater symmetry between the NM_H_ and NM_L_. Although there is an imbalance in symmetry between the NM_H_ (2.10 V) and NM_L_ (2.03 V), the characteristic of exceeding 2 V can still effectively overcome the noise variations in the unskewed monolithic inverter with β of 25.1. Figure 6 shows the V_out_—V_in_ dynamic characteristics of the monolithic inverter with β of 25.1. The rising time of 4.9 μs and the falling time of 3.2 μs indicated that the monolithic inverters could operate at a higher switch speed.

**Figure 7:**
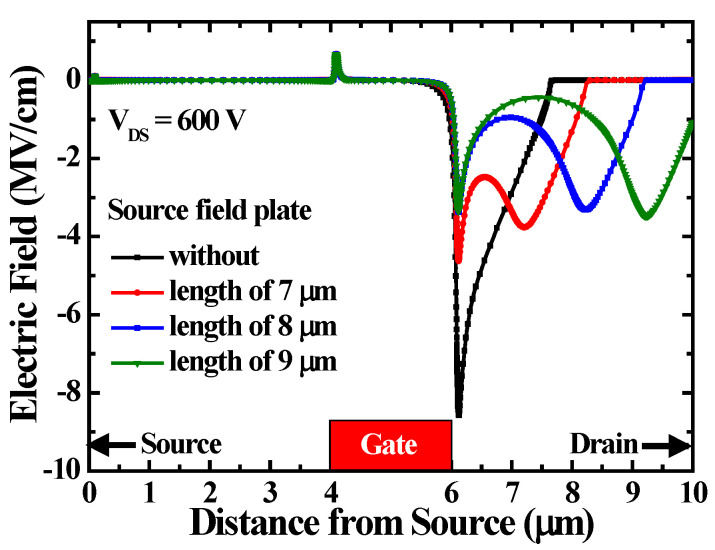
Simulated electric field distributions without and with various lengths of source field plates.

To explore the dependence of the breakdown voltage on the SFP length, the breakdown voltages of the various monolithic inverters were measured. Compared with the breakdown voltage of 746 V without an SFP, breakdown voltages of 780 V, 855 V, and 821 V were obtained by incorporating 7, 8, and 9 μm-length SFPs, respectively. It is worth noting that the breakdown voltage was improved by incorporating the SFP. However, the improved breakdown voltage was still affected by the SFP length. To explore the simulated electric field distribution along the 2DEG channels using various-length SFPs, simulations were carried out using the NTU DDCC TCAD platform, where the Poisson and drift–diffusion equations were solved self-consistently with a two-dimensional finite element method (2D FEM). Figure 7 shows the simulated electric field distributions without and with 7, 8, and 9 μm-length SFPs operating at a V_DS_ of 600 V. The simulated electric field profiles show that MOS-HEMTs without an SFP exhibit a pronounced peak near the gate electrode, which makes them more prone to premature breakdown. Introducing an SFP redistributes the electric field and reduces the peak electric field intensity, thereby enhancing breakdown voltage. The impact of SFP length is equally critical in determining the electric field distribution and breakdown voltage. The simulations show that a 7 µm SFP, although capable of reducing the gate-side peak compared to the case without the SFP, still produces significantly high residual peaks. This is because the SFP is too short to sufficiently spread the potential toward the drain, leaving the electric field concentrated near the gate region. In contrast, the 8 µm field plate achieves the most balanced redistribution. The first peak near the gate is effectively suppressed, and the second peak toward the drain side remains moderate. As a result, the 8 µm design consistently yields the highest breakdown voltage. When the SFP length is further increased to 9 µm, the redistribution becomes excessive, pushing the depletion region too close to the drain contact. This leads to a re-enhanced local peak at the SFP edge, which can even exceed the values observed in the 7 µm case, thereby reducing the effectiveness of breakdown improvement.

## 4. Conclusions

In this study, D- and E-mode GaN-based 120 nm-wide fin-gated multiple nanochannel MOS-HEMTs were fabricated on the epitaxial double-2DEG channel layers. To explore the affected functions and characteristics, various-length SFPs were introduced into the D- and E-mode MOS-HEMTs. By integrating D- and E-mode MOS-HEMTs, the monolithic inverters with CMOS-HEMT architecture were obtained. To control the drain-source current of the D-mode MOS-HEMT to obtain a β value of 25.1 in monolithic inverters, the performance of unskewed inverters was achieved. In the unskewed inverters, the NM_L_ and NM_H_ were 2.03 V and 2.10 V, respectively. It indicated that the unskewed monolithic inverters could tolerate noise variations of more than 2 V. Furthermore, the dynamic experimental results, based on a rising time of 4.9 μs and a falling time of 3.2 μs, verified that the unskewed monolithic inverter could operate at high switch speed. Using the TCAD simulation, the highest breakdown voltage was achieved when the SFP edge was located at the center between the gate and the drain. It was found that the unskewed monolithic also could work at a high breakdown voltage of 855 V. Table 2 lists the comparison results of inverters [23,24,25,26,27,28,29]. The studied monolithic inverters have promising superior characteristics. Although the impact of the SFP on the monolithic inverters was simulated and demonstrated, the structure and dimension of the optimal SFPs based on the thickness and properties of the passivation layer and the structure and geometry of the D- and E-mode MOS-HEMTs are still being simulated and designed in detail. In this study, the performance of the SFP incorporated monolithic inverters was demonstrated. To achieve optimal unique performance for various monolithic inverters, different SFP structures are expected. In this study, the functions and improved characteristics of source field plates are incorporated into the monolithic inverters. Because specific characteristics of the source field plate, gate field plate, drain field plate, and multiple field plates can improve the different characteristics. Therefore, by standing the different geometries and various field plates in the monolithic inverters, the characteristics of the resulting monolithic inverters can be enhanced.

## Figures and Tables

**Table 1:** Characteristics of monolithic inverters with various β values.

β	V_IL_ (V)	V_OL_ (V)	V_IH_ (V)	V_OH_ (V)	V_OS_ (V)	NM_L_ (V)	NM_H_ (V)	V_out_ = V_DD_/2 V_in_ (V)
18.0	2.20	0.13	2.95	5.0	4.87	2.07	2.05	2.61
25.1	2.12	0.08	2.90	5.0	4.91	2.03	2.10	2.51
36.2	2.05	0.07	2.82	5.0	4.93	1.98	2.20	2.45

**Table 2:** Comparison data of inverters.

Parameter		NM_L_ (V)	NM_H_ (V)	V_out_ =V_DD_/2 V_in_ (V)	Ref.
Material *					
n-MoS_2_(D), p-WSe_2_(D)		1.407	1.305	~1.6	[23]
n-IGZO(D), p-Si(E)		1.34	1.22	~1.6	[24]
n-MoS_2_(E), p-MoS_2_(E)		1.95	2	2.49	[25]
n-ITO(E), p-Te-TeO_x_(D)		0.69	1.03	1.18	[26]
n-MoTe_2_(E), p-MoTe_2_(E)		1.08	1.98	~1.7	[27]
n-WSe_2_(E), p-WSe_2_(E)		0.7	0.98	~0.9	[28]
n-WS_2_(E), p-WSe_2_(E)		1.23	1.35	0	[29]
GaN-based (D)(E)		2.03	2.10	2.51	this work

* D: D-mode and E: E-mode.

## Data Availability

The original contributions presented in this study are included in the article. Further inquiries can be directed to the corresponding author.
